# Cor*Deep* and the Sacrobosco Dataset: Detection of Visual Elements in Historical Documents

**DOI:** 10.3390/jimaging8100285

**Published:** 2022-10-15

**Authors:** Jochen Büttner, Julius Martinetz, Hassan El-Hajj, Matteo Valleriani

**Affiliations:** 1 Max Planck Institute for the History of Science, Boltzmannstr. 22, 14195 Berlin, Germany; 2 BIFOLD—Berlin Institute for the Foundations of Learning and Data, 10587 Berlin, Germany; 3 Institute of History and Philosophy of Science, Technology, and Literature, Faculty I—Humanities and Educational Sciences, Technische Universität Berlin, Straße des 17. Juni 135, 10623 Berlin, Germany; 4 The Cohn Institute for the History and Philosophy of Science and Ideas, Faculty of Humanities, Tel Aviv University, P.O. Box 39040, Ramat Aviv, Tel Aviv 6139001, Israel

**Keywords:** sphaera, object detection, historical illustrations, digital humanities, artificial intelligence, dataset

## Abstract

Recent advances in object detection facilitated by deep learning have led to numerous solutions in a myriad of fields ranging from medical diagnosis to autonomous driving. However, historical research is yet to reap the benefits of such advances. This is generally due to the low number of large, coherent, and annotated datasets of historical documents, as well as the overwhelming focus on Optical Character Recognition to support the analysis of historical documents. In this paper, we highlight the importance of visual elements, in particular illustrations in historical documents, and offer a public multi-class historical visual element dataset based on the *Sphaera* corpus. Additionally, we train an image extraction model based on YOLO architecture and publish it through a publicly available web-service to detect and extract multi-class images from historical documents in an effort to bridge the gap between traditional and computational approaches in historical studies.

## 1. Introduction

The current study of the vast amount of unexplored sources available in archives and libraries by means of classical historical methods remains beyond our capabilities. This led to the emergence, in the recent years, of a large number of initiatives aimed at digitizing such collections of historical documents, such as the Google Book Search (GBS) and Open Content Alliance (OCA) [1]. These digitization efforts have bestowed on us a wealth of historical material in digitized format. The vast majority of these digital copies consist of raster images from flat bed scans or page photographs. While such raster images are abundant, their content, i.e., text and illustrations, are generally non–machine readable, and thus remain hidden to the readers until they open and read the digital copy.

In the frame of the project *The Sphere: Knowledge System Evolution and the Scientific Identity of Europe* (https://sphaera.mpiwg-berlin.mpg.de/, accessed on 2 October 2022) we analyze a collection of 359 editions centered on the *Tractatus de sphaera* by Johannes de Sacrobosco (–1256) and printed between 1472 and 1650. This collection, dubbed the *Sphaera* Corpus, contains around 76,000 pages with 30,000 visual elements. The elements are separated into four distinct categories based on their function within the printed book, as discussed in Section 3. These visual elements were manually collected and labelled by student assistants, which allowed us to construct a relatively large and unique mutli-class dataset. As a result, we were able to use this dataset to train a neural network to detect and classify visual elements from the *Sphaera* Corpus, then to test its performance on other corpora. Given the success of our model, as reported in Section 4 and Section 5, and its ability to change the way we analyze historical sources, we integrated it in a public web-service in an effort to reach a wide audience of historians regardless of their level of computer literacy. In this respect, our contributions in this paper can be summarized as follows:

1 Highlighting the importance of the study of visual elements in historical corpora.
2 Providing a curated dataset for the detection of visual elements in historical corpora (accessible here [2]) and comparing the performance of object detection models on this *real* dataset as opposed to large synthetic historical datasets.
3 Proposing a dedicated object detection model for visual elements in historical documents, and comparing it with state-of-the-art historical page segmentation methods.
4 Providing an easy to use web-service (https://cordeep.mpiwg-berlin.mpg.de/, accessed on 2 October 2022) for the detection of visual elements in historical documents.

In the following, we introduce the current approaches to historical document page segmentation and image extraction in Section 2, and introduce the *Sacrobosco* Visual Elements Dataset (S-VED) derived from the *Sphaera* Corpus in Section 3. We then evaluate the performance of three different model architectures, YOLO, DocExtractor, and Faster-RCNN, on S-VED in Section 4, after which we evaluate the generalization abilities of these models on various datasets, as well as whether training on S-VED results in a better model than large synthetic datasets in Section 5. Finally, we present our web-service in Section 6, and discuss, in Section 7, some of the limitations encountered while building S-VED and when training and evaluating the results of models trained on this dataset.

## 2. State of the Art

The availability of large amounts of digitized historical documents opened the door to the use of computational approaches for their analysis [3]. While research in the field of historical document processing is diverse, ranging from manuscript dating and writer identification to building large digital libraries, the main bulk of scholarship in this regard was devoted to Optical Character Recognition (OCR) approaches in an effort to extract and automatically read the text in raster images of historical sources. Initial efforts in this field relied on simple computer vision techniques for image segmentation and word and letter extraction [4]. However, the variability of fonts and page layouts of historical documents, as well as the large variety of alternative spellings cause a high number of errors. With the onset of deep learning, OCR research adopted neural network architecture to achieve its objectives. Pre-processing approaches aimed at page segmentation as a preparatory step before OCR analysis relied on architectures such as U-Net [5] and Mask R-CNN [6], similar to those presented in [7,8]. This step is often followed by approaches such as [9,10,11] which rely on a combination of Convolutional Neural Networks (CNN) and Recurrent Neural Networks (RNN) to extract features and process texts.

While advancement in the domain of text recognition in historical documents is clear, the same cannot be said of the field of image recognition within these documents, which is often considered a pre-processing step during page layout analysis [3]. This is all the more remarkable if we consider that historical disciplines have become increasingly concerned with the relationship between text and visual elements, with an ever-increasing number of historical projects dedicated to this subject [12,13,14]. While page segmentation approaches, such as [7], return text/non-text masks, the latter of which often includes visual elements, this remains insufficient for any meaningful historical study, as such approaches often lack accurate visual element localization, as well as semantic classification of these elements. In this regard, one of the main hurdles hindering the success of semantic visual element recognition within historical documents is their high variability, as well as the general scarcity of coherent historical datasets focused on visual element recognition, with only 11 out of the 56 historical document datasets mentioned in [15] containing graphical elements. Additionally, in the majority of cases where visual elements were recorded in these datasets, they were not classified according to their semantic classes [16].

This high variability of visual elements means that some of the available datasets concern a specific time period or a specific medium, such as hand-written manuscripts, printed books, or newspapers. This is clearly the case of the Newspaper Navigator Dataset [17], which contains visual content from 16 million historic newspapers in the United States published between 1798 and 1963, or the HORAE dataset [18], which focuses on books of hours from the late Middle Ages. In response to the scarcity of historical datasets, synthetic datasets emerged in an effort to create larger, more diverse, and highly variable datasets without resorting to manual annotations. Such synthetic datasets were proposed in [19], which offers DocCreator, a platform to automatically generate synthetic, historically looking document images, while [8] created a synthetic dataset from elements of a large corpus of *real* historical-document images that helped in training an accurate historical document segmentation model. Refs. [10,20], moreover, also relied on synthetic data to enhance the performances of their OCR models for printed and handwritten documents. In this paper, we present and rely on a hand-curated dataset of historical illustrations from the early days of printing to train a well performing object detection model and highlight the need for larger datasets that cover the period-specific high variability of visual elements.

## 3. The *Sacrobosco* Visual Elements Dataset (S-VED)

The importance of visual elements in the *Sphaera* Corpus stems from the nature of the corpus’s editions, which have already been analyzed from very different perspectives [21,22,23,24,25,26]. The 359 *Sphaera* editions, centered on the *Tractatus de sphaera* by Johannes de Sacrobosco (–1256)and printed between 1472 and 1650, were primarily used to teach geocentric astronomy to university students across Europe, generally between fourteen and eighteen years old [27]. Their visual elements, therefore, played an essential role in visualizing the ideas, messages, and concepts that the texts transmitted. This high inter-connectivity and dependence between text and visual elements implies that the latter are integral to the comprehension of the former and vice versa. As a precondition for studying the relation between text and visual elements, a time-consuming image labelling process was conducted by five student assistants in order to extract and label the visual elements from the 76,000 pages of the corpus. These students were instructed to create a bounding box around each visual element created by a single woodblock and assign it to a single class (see Section 7 for difficulties faced during the labelling process and their effect on the model output). Each students worked, on average, around 40 hours per month and completed the labelling work in about 5 months. This well curated data represents a dataset of visual elements of early modern scientific works, and allows us to train a model to extract images from similar corpora.

**Figure 1:**
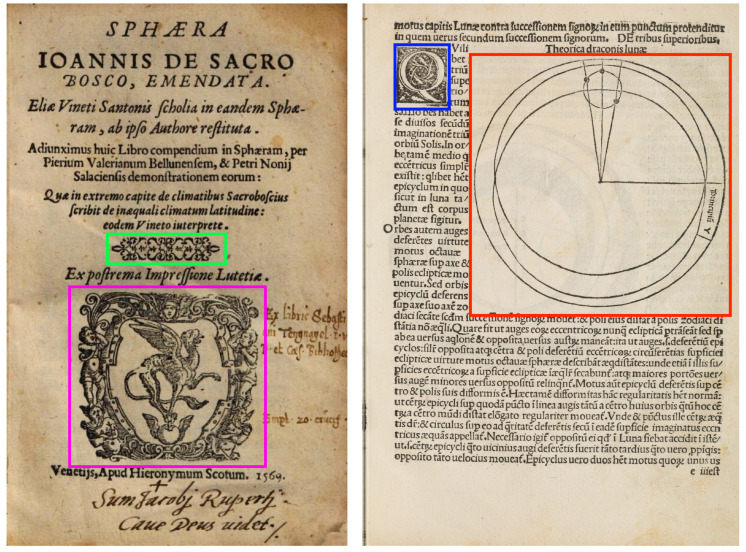
Two pages from the S-VED showing the four different classes. Green: Decoration, Magenta: Printer’s Mark, Blue: Initial, and Red: Content Illustration (Left page: *Sphaera Ionnis de Sacro Bosco*, 1569, Venice, https://hdl.handle.net/21.11103/sphaera.101040, accessed on 2 October 2022, Österreichische Nationalbibliothek. http://data.onb.ac.at/rep/1089F5CC, accessed on 2 October 2022 —Right page: *Sphaerae mundi compendium foeliciter inchoat*, 1490, Venice, https://hdl.handle.net/21.11103/sphaera.100885, accessed on 2 October 2022, courtesy of the Library of the Max Planck Institute for the History of Science).

The work of the student assistants resulted in the creation of the Extended *Sacrobosco* Visual Elements Dataset (S-VED${}_{extended}$). This dataset consists of almost 30,000 visual elements located on 23,190 pages, covering almost 30% of the corpus’s pages, and classified into four classes whose count is shown in Table 1. These classes represent the four main categories of visual elements found in the *Sphaera* Corpus (Figure 1). However, copyright constraints prevent us from publicly sharing many of the 23,190 pages of the S-VED${}_{extended}$. To circumvent this issue, we created the S-VED (accessible here [2]) , which contains a total of 4000 pages sampled from the S-VED${}_{extended}$ and shares its distribution (Table 1).

The four classes are:

1 **Content illustrations**: visual elements inserted in and around the text in order to explain, enrich, describe, or even criticize the content of the latter.
2 **Printer’s Marks**: visual elements often located at the beginning or the end of the book, and are considered to be the emblems, or insignia, of the printers who produced the books in question.
3 **Decorations**: decorative items placed on the page for multiple purposes, e.g., marking the end of a paragraph or chapter, or simply decorating the contour of the text.
4 **Initials**: small visual elements each representing a letter at the beginning of a paragraph. These letters were often abundantly decorated, and served to highlight the structure of the text.

**Figure 2:**
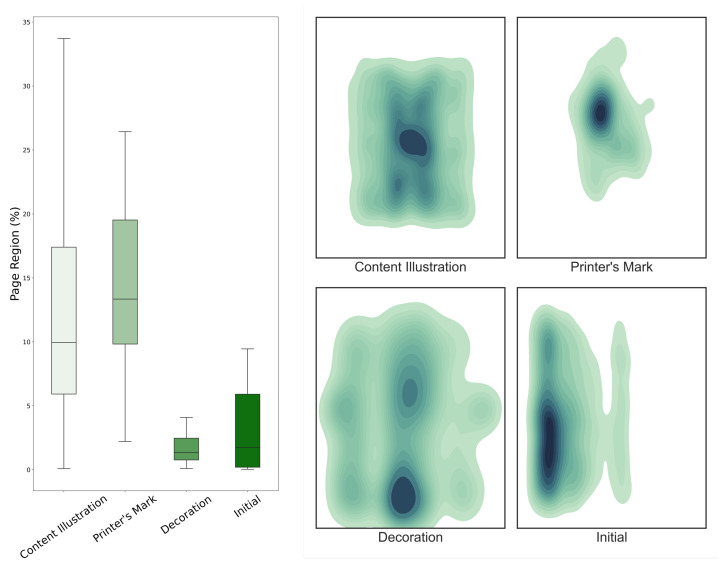
Distribution of visual element classes by page size percentage (90% interquartile range) and bounding box centroid localization.

The largest component of both S-VED and S-VED${}_{extended}$ is the Content Illustration class, which constitutes almost 70% of the entire dataset. The visual elements of this class are those that convey the scientific information, as they are generally placed within a treatise’s text and represent its content. These images vary in size, with the majority covering between 7 and 17% of a page’s area, while their position varies across the page but generally avoids the margins (Figure 2). As this section of the dataset concerns the scientific content of the editions under investigation, it is evident that this collection is particularly relevant for the history of astronomy on the one hand and the study of the evolution and transformation of visual language in science on the other.

The number of identified Printer’s Marks is smaller since each edition has either none, one, or a maximum of two. Since these images are missing in some cases, the number of Printer’s Marks is lower than the total number of editions in the *Sphaera* Corpus. Visual elements of this class are the largest in size within the discussed datasets because they were often printed on their own dedicated page, frequently placed in the center with no regard for space constraints (Figure 2). Printer’s Marks convey relevant information for book historians, particularly those involved in the economic history of the book. The oldest editions of the corpus were printed in 1472, less than only 20 years after the publication of Gutenberg’s Bible. The 178 years covered by the corpus, therefore, represent the period during which the book as a commercial product evolved in conception and design to resemble the book as it is known today. The presence or absence of a Printer Mark in an edition alone denotes the awareness of the printers and publishers of the time about their fundamental role in promoting or hindering the dissemination and success of specific scientific content [26]. Moreover, the Printer’s Marks’ emblems are rich figurative illustrations, dense in details that are never random or merely the result of artistic taste. For large-scale editorial initiatives, for instance, when the amount of capital required for the production was particularly relevant, printers often joined forces (and money) and divided the investments and profits among themselves. In such cases, *ad hoc* joint ventures were signalized to the audience by a Printer Mark that displayed elements and details of the Printer’s Marks of the individual printers and publishers who joined the project. Lastly, Printer’s Marks, or the details of which they were comprised, were often used to signal the closeness of the printer’s workshop to a specific societal group or well-established institutional network. These could be religious orders, specific confessions, a single city and its ruler, or philosophical schools [28,29,30,31,32,33].

The visual elements of the Decoration class generally occupy the least page space but can be found almost throughout the whole page area, including the margins. Initials are small in size and are predominantly located along the left-hand side of the page, which is to be expected given that *Sphaera* Corpus texts are written in left-to-right scripts, such as Latin, French, Italian, and German, among others (Figure 2). Initials and Decorations and book ornaments, in general, are collections that are useful for understanding the framework of book and printing history. Decorations are studied in reference to the patterns and stylistic choices they display. It is estimated that about one-third of the print runs printed during the early modern period bear no imprint at all; therefore, the study of Decorations can be particularly useful for determining the printers of such books. Moreover, as the majority of visual elements in early modern books were the result of the woodblock printing technique and woodblocks themselves were exchanged among printers, Decorations can reveal cooperative networks of economic nature among printers.

The purpose of studying Initials is two-fold. The first is very similar to the one described for the Decorations. Namely, Initials are large-sized letters that mostly display a sophisticated graphic for the font, and are printed over a decorative background, usually showing very elaborate patterns and ornaments. Secondly, if collected from a corpus of great size, Initials help us to understand one aspect of the evolution of the book as a commercial product. This aspect is related to the structure of the texts. Before the advent of printing technologies, manuscripts were assemblages of texts collected over long periods of time. Over generations, manuscripts often changed owners; consequently, the individual and almost personal aspects a manuscript exhibited were dependent upon the current owner and the way they used the manuscript. In this context, large-size Initials used throughout the body of the text were extremely useful for quickly identifying when a new text or a new argument within one manuscript began. Though commonplace today, title pages, chapter numbers, or similar structural aspects of books were not used or extremely rare. Initially, printed books were mostly conceived and designed similarly to the well-known form and format of manuscripts. While the printed book became a product for a larger and more “anonymous” audience, its layout evolved, and title pages, headings for texts, chapters, sections, as well as running heads and other components began to appear, improving and generalizing the usability of books. During this stage, Initials began losing their function and increasingly assumed a merely ornamental function, eventually rendering them largely obsolete. A statistical study concerning Initials that covers an extended time period can, therefore, reveal the major phases of this process [34,35,36,37,38].

This classification into different semantic classes was only possible due to rigorous historical analysis. However, other datasets (shown in Section 5) do not distinguish between illustration classes, and lump them into a single category of visual material. This led us, in some cases, to merge our four classes into one in order to compare our results with those of other datasets. We additionally collected an equal number of negative examples, i.e., pages without any visual elements, that were randomly sampled from the corpus. In total, the S-VED${}_{extended}$ contains 46,380 pages along with the visual element annotations, which we use to train our model as shown in the following sections. The model, moreover, is publicly available through our dedicated web-service (Section 6).

## 4. Detecting Visual Elements in the *Sphaera* Corpus

The time-consuming creation of the S-VED opened the door towards the automatic detection of these visual elements, which would be beneficial not only within the scope of the *Sphere* project, but also for other projects that deal with similar corpora. With this aim in mind, we trained three different deep learning–based object detection models on the visual elements of the *Sphaera* Corpus. We applied and compared the state-of-the-art object detector YOLOv5 [39], the pixel-wise segmentation classifier DocExtractor, a state-of-the-art off-the-shelf system for element extraction from historical documents [8], and Faster-RCNN, a two-step object detector [40].

### 4.1. Models and Training

**YOLO** [41] is considered a state-of-the-art object detection system both in regard to speed and accuracy, which prompted us to use it for the detection of graphical elements in historical documents. YOLO is an abbreviation for “You Only Look Once” and describes the algorithm’s ability to detect objects in images with a single forward pass through its network. In this paper, we use the YOLOv5, which is the fifth and latest version of this neural architecture. YOLO’s neural network can be described in three stages: The first consists of a backbone that extracts features from the input, after which comes a neck that aggregates the features, and finally a head that detects objects. The backbone could be substituted by any neural network architecture. However, YOLOv5 provides five predefined such architectures; they differ in size, and thus in speed and performance. In this case, we used the so-called YOLOv5l architecture, which corresponds to the second largest available architecture and offers robust feature extraction but still fulfills our inference speed requirements. Our YOLOv5 model was initialized with the pretrained weights used on the COCO [42] dataset, and trained with an initial learning rate of 0.01. At training time, various data augmentation techniques were applied to prevent overfitting to the training data, which include standard approaches such as affine transformations and color augmentations, as well as perspective and mosaic augmentations. These account for the scan angle differences and object sizes, respectively.

**DocExtractor** is a line-level page segmentation system introduced by [8], which generates pixel masks for both visual elements and text in historical documents. DocExtractor’s architecture relies on an encoder-decoder (namely a modified U-Net [43] with a ResNet-18 [44] encoder) for pixel-wise segmentation. We trained this “out-of-the-box” network on our data using the recommended hyper-parameters (https://github.com/monniert/docExtractor, acccessed on 2 October 2022) and used it to benchmark our YOLO model, as it has specifically been proposed for processing historical documents and because its architecture is commonly used in state-of-the-art OCR systems [45] to segment pages and extract text regions, outperforming Mask-RCNN [6] as shown in [8].

**Faster-RCNN** is a two step object detection model, composed of a Region Proposal Network (RPN) and a detection network [40]. The RPN is composed of a fully-convolutional network, trained end-to-end to generate region proposals which are fed to the object detection model. We used a ResNet50 as the backbone, and following our approach with the YOLO model, initialized the model with weights pretrained on COCO [42], and fine-tuned it with an initial learning rate of 0.005.

These models are trained on the S-VED${}_{extended}$ training-split, which contains a total of 38,426 page images of which 3772 are used for validation, while the test-split contains 7954 page images. The S-VED split consists of the same validation and test split, however, it only uses a subset of 4000 page images from S-VED’s training split as its training set. Almost half of the samples in every split contained a visual element, while the other half was a negative sample. To account for the general variability of image sizes, especially those of scanned books, we trained the two above-mentioned models using large and small S-VED/S-VED${}_{extended}$ image sizes: 1280 × 1280 and 640 × 640, respectively. The models were trained on a NVIDIA A100GPU until no significant performance increase was observable, which took 35 epochs (equivalent to a single compute day) for the YOLOv5 model, 25 epochs for DocExtractor, which took approximately a week, and 40 epochs for Faster-RCNN, which required 36 h. The significantly greater training time of DocExtractor stems from the fact that the “out-of-box” DocExtractor training pipeline only supports a batch size of 1, originating from the memory restrictions the authors of DocExtractor were bound to.

### 4.2. Model Evaluation

To evaluate the performance of the above-mentioned models in Section 4.1, we rely on the commonly used Intersection over Union (IoU) metric, whose values are between 0 and 1, to assess the accuracy of our bounding box localization (see Equation (1)):

(1) $$IoU=\frac{B_{GT}\cap B_{P}}{B_{GT}\cup B_{P}}$$

where $B_{GT}$ denotes the area of the ground truth bounding box and $B_{P}$ denotes the area of the predicted bounding box. A low IoU value indicates a detection that does not sufficiently overlap with our ground truth bounding box, while a high IoU value indicates a higher overlap between the predicted and ground truth bounding boxes. Following [46], we set a threshold of 0.5, above which a detection is considered correct if the predicted class is also correct. From these correct detections, we report the average precision (AP) values in Table 2 for the two input sizes on both S-VED and S-VED${}_{extended}$, where the AP is calculated by averaging the precision values corresponding to all recall values between 0 and 1 using an “all-point interpolation” technique [46]. In the calculation of the AP, empty pages (pages that do not include an illustration) can only contribute in a negative manner. In other words, false positive illustrations on empty pages penalize the AP, whereas pages that are correctly recognized as devout of visual elements by a model do not improve the AP score. We emphasize this fact due to the high number of empty pages in our test set. The AP values in Table 2 show the evaluation of all our tested models on a single, comprehensive, visual element class containing all four classes discussed in Section 3. The reason for this merger is due to the fact that an “out-of-box” DocExtractor is not designed to differentiate between the four categories presented in the S-VED. As evident from Table 2, YOLO outperforms both DocExtractor and Faster-RCNN by varying degrees. The high AP scores achieved on both high and low resolution images can be considered an almost perfect result. The small variations—in the order of 10${}^{-2}$ and 10${}^{-3}$—between the AP scores of the two different input sizes is investigated using GradCAM [47], and is generally attributed to our image labelling strategy (see Section 7). This is particularly noticeable where the models consider a group of visual elements in a page as a single object in lower resolution inputs, but consider the same group as multiple objects with higher input resolution, thus directly affecting the AP scores. When it comes to the training dataset, the results of training YOLO, DocExtractor, and Faster-RCNN on both S-VED and S-VED${}_{extended}$ prove to be similar, with an almost insignificant drop in AP when switching from the latter to the former. This indicates that S-VED, while smaller than the *extended* version, is still highly useful to train object-detection models aimed at historical visual elements in printed books.

Since the best YOLO AP values for both S-VED and S-VED${}_{extended}$ were obtained with an input image resolution of 640 × 640, and those of Faster-RCNN with 1280 × 1280, all further evaluations in this paper will be reported using the aforementioned image resolutions for their respective models.

**Figure 3:**
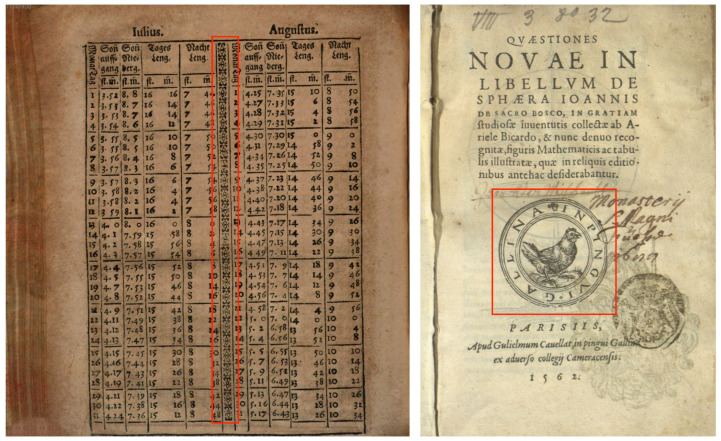
Classification Errors. (**Left**) False Negative: the model failed to recognize a decoration visual item embedded within a numerical table (*Sphaera*, 1614, Leipzig, https://hdl.handle.net/21.11103/sphaera.101208, accessed on 2 October 2022, Bayerische Staatsbibliothek. urn:nbn:de:bvb:12-bsb11110900-6); (**Right**) Class misclassification: the model classified the image as a Content Illustration while the image is a Printer’s Mark printed at the beginning of the book on a dedicated page (*Qvaestiones novae in libellvm de sphaera Ioannis de Sacro Bosco*, 1562, Paris, hdl.handle.net/21.11103/sphaera.100156, accessed on 2 October 2022, Universitätsbibliothek Augsburg. urn:nbn:de:bvb:384-uba003411-4).

**Figure 4:**
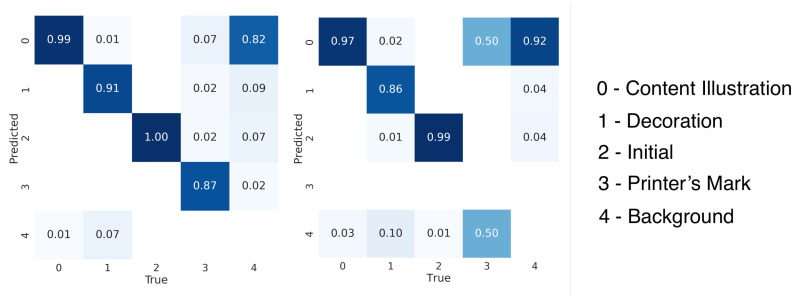
Class confusion tables for (**Left**) YOLOv5 (S-VED${}_{extended}$, 640 × 640, confidence threshold = 0.2), (**Right**) Faster-RCNN (S-VED${}_{extended}$, 1280 × 1280, confidence threshold = 0.7).

In order to further investigate the precision of the YOLO and Faster-RCNN models on the four different classes discussed in Section 3, we report their AP results in Table 3. Both YOLO and Faster-RCNN record their highest AP for Content Illustration and Initial classes for both S-VED and S-VED${}_{extended}$, while this average precision drops for Printer’s Marks and Decorations, with varying degrees, for both models on the tested datasets. While the drop in average precision can be generally related to the significantly lower number of Decorations and Printer’s Marks in both S-VED and S-VED${}_{extended}$, we investigate the source of this drop by looking at error cases in Figure 3, as well as the Confusion Matrices shown in Figure 4. It is clearly visible in this case that the lower average precision for the Decoration class in both models’ results is due to a high number of False Negatives signaled by the relatively high percentage of instances missed by the model and, consequently, classified as background (see Figure 3: Left). On the other hand, the lower average precision reported for the Printer’s Mark results stems from False Negatives due to inter-class misclassification, where the Printer’s Marks are often classified as Initials, Decorations, and most frequently Content Illustrations, especially in the Faster-RCNN results. This inter-class misclassification is due to the fact that Printer’s Marks are often very similar in form to Content Illustrations (see Figure 3: Right), but generally differ from the latter semantically. While Printer’s Marks are commonly placed at the beginning or end of the book and identify the printer(s) who produced the book, Content Illustrations are exclusively located within the inner pages of the book. Since page information is not provided to the model as an input, this inter-class misclassification is expected. A detailed look at the limitations and fringe cases that cannot be effectively solved by this model is presented in Section 7. Overall, the YOLO model trained on S-VED${}_{extended}$ makes 436 false positive detections from a total of 4511, whereas for the Faster-RCNN model trained on S-VED${}_{extended}$, this ratio is 671 to 4749. The confidence thresholds for the two models were chosen to maximize the average between precision and recall, which resulted in a confidence threshold of 0.2 for YOLO and 0.7 for Faster-RCNN.

**Figure 5:**
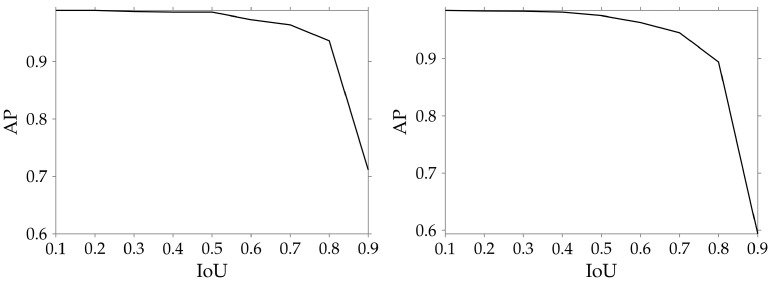
Average precision (AP) results with different IoU detection thresholds on *sphaera* data for YOLOv5 (S-VED${}_{extended}$, 640 × 640) (**left**) and for Faster-RCNN (S-VED${}_{extended}$, 1280 × 1280) (**right**).

Finally, we evaluate the localization capabilities of both YOLO and Faster-RCNN models by plotting the variation of the mAP with different IoU thresholds as shown in Figure 5. It is clearly visible that the mAP remains above 0.9 up to an IoU threshold of 0.8 in both cases, which indicates that the predicted bounding boxes are well localized with respect to the ground truth boxes.

The results above clearly indicate that dedicated object detection models, such as YOLO and Faster-RCNN, are better suited to the specific task of visual element detection. However, while DocExtractor lags behind these two models when it comes to object detection, the former is designed as a page segmentation model and is thus able to return, in addition to image regions, line segmentation results that could also be useful for scholars. Given our focused interest in visual element detection in historical corpora, the next section is dedicated to evaluating the generalization ability of the better performing model, YOLO, on numerous *real* and synthetic datasets.

## 5. Generalization to Other Historical Corpora

Having shown that YOLO trained on either S-VED or S-VED${}_{extended}$ (YOLO${}_{S-VED}$ and YOLO${}_{S-VEDX}$ for short) performs better than Faster-RCNN, as well as other state-of-the-art pixel segmentation approaches for visual element extraction trained on the same data (namely DocExtractor), we evaluated the generalization abilities of the YOLO models on multiple datasets containing historical documents from different periods and topics. However, one of the major setbacks in applying Machine Learning to historical documents is the relative lack of large, labelled, and open source historical datasets compared to other fields, e.g., autonomous driving, robotics, etc. Despite this lacuna, generally discussed in Section 2, we highlight below several relatively small historical datasets on which we test the generalization abilities of our model in order to compare the performance of YOLO trained on S-VED and S-VED${}_{extended}$ to that trained on a large synthetic historical image dataset, SynDoc [8], which was created to remedy the shortage of *real* historical datasets discussed above.

### 5.1. Datasets Used

**Mandragore dataset** is offered by the Bibliothèque Nationale de France [48], is a collection of illuminated manuscripts, which are handwritten manuscripts whose pages are often heavily decorated with painted illustrations dating mainly to between the 11th and 15th centuries. The dataset contains annotated pages from 8 different manuscripts totalling 1691 pages.

**RASM dataset** is a dataset of a 100 historical Arabic pages including both text and visual elements [49]. The RASM dataset not only differs from the S-VED in the fact that the former is mostly composed of handwritten manuscripts with hand-drawn drawings and diagrams while the latter is composed of printed pages and visual elements from printing woodblocks. It differs also because the language of the RASM manuscripts is Arabic, while the S-VED dataset contains mostly Latin sources.

**IlluHisDoc dataset** was presented in [8] as a benchmark for historical document illustration segmentation. This dataset contains 400 annotated pages from 20 historical documents from the Bibliothèque Nationale de France ranging from pre-printing manuscripts to 19th century printed books, distributed across four main document classes: printed documents with multiple illustrations such as drawings, decorations, paintings, and photos (P); manuscripts with scientific diagrams (MSS); manuscripts with illuminations (MSI); and manuscripts with drawings (MSD).

***Pseudo Proclus*****Visual Element Dataset** is created within the *Sphere* Project and semantically resembles the S-VED. Pseudo Proclus’s *Sphaera* is a text that was first printed in 1499 in Venice and is similar to Sacrobosco’s *Sphaera*, which is the basis for the S-VED. Both of these texts were aimed at university students and discuss the topic of geocentric astronomy in the early modern period [50]. However, their visual apparatuses, while portraying similar topics, do not entirely overlap. This dataset contains a total of 2213 visual elements—across 74 editions—divided into the same classes as S-VED.

**SynDoc dataset** is a synthetic document dataset introduced in [8] to tackle the lack of large-scale annotated historical document datasets. SynDoc contains a total of 10,000 page images generated from a randomized page generation engine that created a large number of page backgrounds and filled them with a combination of text, images, drawings, and glyphs from multiple sources. The result is a dataset that contains a large number of images that mimic the layout and content of a wide range of historical documents.

The four historical datasets (Mandragore, RASM2019, illuHisDoc, and Pseudo Proclus) combine a wide range of page layouts from printed and handwritten sources across a long period of time. They also contain a very diverse repertoire of visual elements, ranging from simple hand drawings to complex illuminated visual elements as well as historical photographs from the 19th century. As a consequence, evaluating our generalization ability on the four historical datasets gives us a good measure of the performance of our model. In contrast, since SynDoc is a synthetic dataset, we refrain from using it to test our model, as it does not represent true historical documents. Instead, we utilize this dataset to train a YOLO model (YOLO${}_{SynDoc}$) with the objective of investigating whether relying on a synthetic dataset is more beneficial than on S-VED, especially when the test set is an out-of-historical-domain corpus. To achieve this, we test YOLO${}_{S-VED}$, YOLO${}_{S-VEDX}$, and YOLO${}_{SynDoc}$ on the *real* historical datasets discussed above, and report their results in Table 4. Additionally, we investigate whether the creation of a hybrid dataset generated by merging elements from diverse *real* historical and synthetic material would result in training a model with better generalization abilities, paving the way for the creation of a large, open source, historical image dataset and image detection benchmark. With this in mind, we iteratively train different models on a combination of four out of the five datasets above, and test it on the unseen dataset, reporting the results in Table 5.

### 5.2. Dataset Evaluation

As can be seen from Table 4, training YOLO on the S-VED${}_{extended}$ dataset yields better results than on SynDoc across the majority of the tested historical datasets in high resolution (1280 × 1280). The only exception appears to be the illuHisDoc dataset, which is the only dataset that encompasses a very large temporal period, including some photographs and modern images that are completely absent from S-VED${}_{extended}$. This in general indicates that the S-VED${}_{extended}$ dataset is more representative of manuscript and early printing visual elements than SynDoc, while the latter might be a better fit for someone looking to exploring a dataset with a very large temporal frame. On the other hand, the results obtained with YOLO${}_{S-VED}$ show high AP scores for both S-VED training sets and Pseudo Proclus datasets in high definition, but fail to produce acceptable results in other historical datasets, underperforming YOLO${}_{SynDoc}$ by almost 0.2 and 0.3 for both RASM and illuHisDoc respectively. This is clearly a result of the lower number of datapoints in the S-VED compared to both S-VED${}_{extended}$ and SynDoc. The situation is generally better when dealing with lower resolution images, where YOLO${}_{S-VED}$ shows comparable or better results than YOLO${}_{SynDoc}$, and only trails the latter by 0.09 in the case of Mandragore, a test dataset which appears to always return low AP scores.

The reason for this perpetually low AP score on Mandragore does not appear to be the high amount of False Positives, but rather the high amount of False Negatives stemming from the fact that decorated Initials, similar to the ones usually annotated in both S-VED (as a separate class) and in SynDoc (along with all other visual elements), are not annotated in the Mangradore ground truth data. The result of this inconsistency is that both models are accurately recognizing these decorated Initials as visual elements, but such detections are considered to be False Positives instead of True Positives. Using YOLO${}_{S-VED}$ and YOLO${}_{S-VEDX}$, which are able to recognize Initials as a separate class, we calculate the AP value while ignoring these Initials, thus avoiding their detection and their effect on the general AP score. By doing this, the AP value increased by values ranging between 0.2 and 0.4 across models and input resolutions, reaching scores comparable to those from other datasets (reported with * in Table 4). The same could not be done with the YOLO${}_{SynDoc}$ since no specific Initials class exists.

Overall both YOLO${}_{S-VEDX}$ and YOLO${}_{SynDoc}$ appear to generalize well to other historical datasets, with the former maintaining an edge over the latter, especially in the case of lower quality input. Additionally, the generalization ability of YOLO${}_{S-VED}$ to other historical datasets appears to be amplified for lower resolution inputs. This generalization, in all cases, comes with a notable drop in AP score. To avoid this, we create multiple hybrid datasets from the combination of the *real* and synthetic datasets discussed in Section 5.1. These hybrid datasets are built by creating a single, comprehensive class of visual elements, since many datasets do not differentiate between the visual element classes as is the case with the S-VED. At each iteration, we train a model on a hybrid dataset composed of a maximum 1000 datapoints—a number chosen to create a diverse hybrid dataset by merging relatively small historical image datasets—from all but one of the datasets mentioned above, and test the performance of that model on the remaining dataset. The results of the various models trained on the different combinations of training sets are reported in Table 5, which shows an expected improvement of the AP scores in the majority of cases, indicative of better generalization abilities. The noticeable increase in generalization when trained with multiple *real* historical datasets serves to emphasize the need for the creation of larger, cross-historical-domain datasets.

## 6. Cor*Deep*: A Web-Service for Detecting Visual Elements in Early Modern Printed Books

Having demonstrated that, in the case of the S-VED, as well as numerous other datasets, YOLO performs better than the current state-of-the-art approaches (see Section 5.2) and driven by our belief in the necessity to generate datasets by extracting and studying visual elements in historical corpora [12,14,51], we provide a public web-service whose sole objective is to extract visual elements from historical corpora (https://cordeep.mpiwg-berlin.mpg.de/, accessed on 2 October 2022) without storing any user data. This web-service is built with the Flask framework and is running on a server of the Gesellschaft für wissenschaftliche Datenverarbeitung mbH Göttingen (GWDG). We rely on the YOLO${}_{S-VEDX}$ model due to its high AP score on the one hand and its ability to classify the detections into four semantically meaningful classes on the other (see Section 3). With this web-service we hope to bridge the gap between the computer scientist and the historian, allowing the latter, without any experience in machine learning, to extract visual elements from large historical corpora with ease.

**Figure 6:**
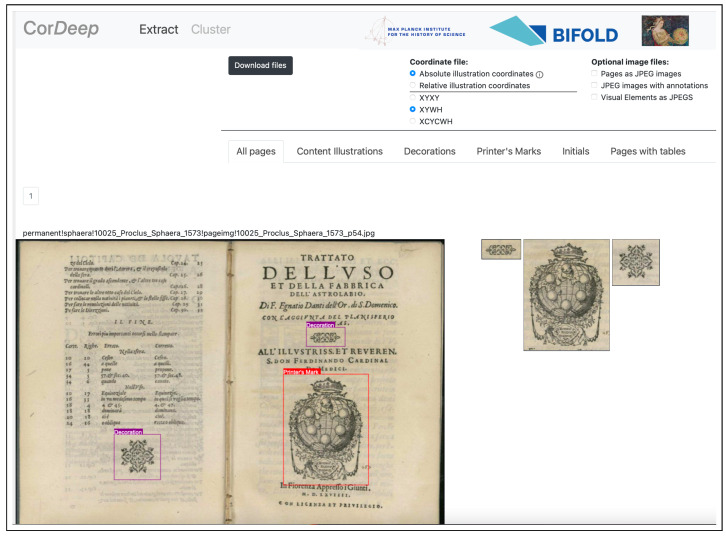
Screenshot of the web application Cor*Deep* depicting the visualization of detected material by the application.

Interacting with the web-service is fairly simple and consists mainly of providing a *historical* document through the standard data upload page. This can be of any format, ranging from a simple PDF, raster images (all common file formats accepted), IIIF manifest JSON files, or by providing a URL to a IIIF manifest JSON file. Once uploaded, the image data is processed on the server by our illustration-extraction model and its output is visualized in the application’s integrated viewer (see Figure 6). This output information can be retrieved in the form of image material or bounding box and label information in a CSV file. While this is a work in progress, historians can expect results along the line of those reported in Section 5.2, showcasing the abilities of YOLO${}_{S-VEDX}$ to extract and classify visual elements in a wide range of historical documents.

## 7. S-VED Limitations

**Figure 7:**
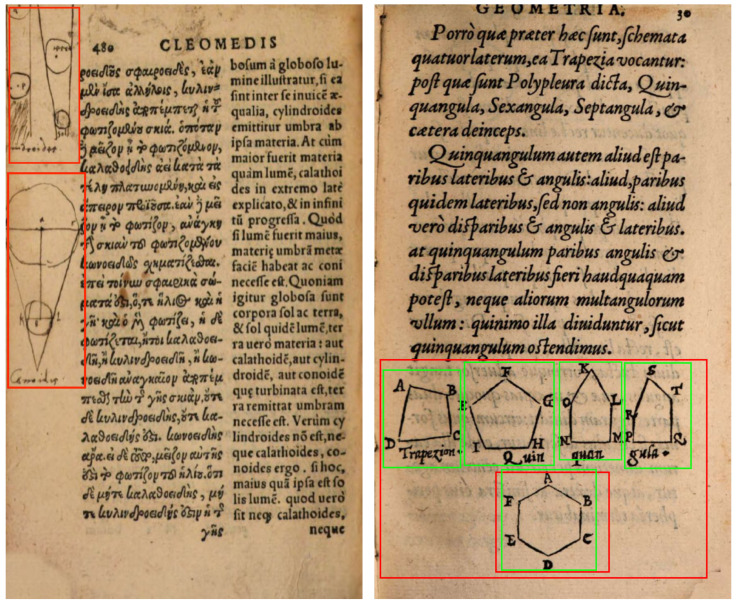
Different types of errors arising from our S-VED annotation strategy. (**Left**) False Positive Content Illustration detection of hand-drawn images in the margins (*Procli de Sphaera Liber I.*, 1561, Basel, München, Bayerische Staatsbibliothek https://hdl.handle.net/21.11103/sphaera.101551, accessed on 2 October 2022. urn:nbn:de:bvb:12-bsb10170643-6); (**Right**) Detection of multiple images as a single visual elements in red, the desired outcome for multiple visual elements in green (*Michael Psellus de arithmetica, musica, geometria*, 1557, Paris, München, Bayerische Staatsbibliothek https://hdl.handle.net/21.11103/sphaera.101497, accessed on 2 October 2022. urn:nbn:de:bvb:12-bsb10205218-2).

While the results of the models presented in both Section 4 and Section 5 are promising, looking at the erroneously classified pages reveals some of the cases that not only remain beyond the reach of our best model, but also force us to discuss what exactly a visual element is. In the S-VED, the focus is solely on printed visual elements; i.e., images, diagrams, initials that were intentionally printed using a woodblock during the book’s production. However, as is clearly visible from Figure 7 (left), the readers of many of these books decided to add their own visual elements, either jotting down their version of a visual element to better explain the accompanying text or adding decorative elements to the margins. While these hand-drawn elements are an interesting research topic on their own, they are not considered to be part of the S-VED. Based on this, some of these hand-drawn images are naturally not detected by our model, but some are and are consequently considered False Positives.

Another issue that we were forced to address during both the labelling and evaluation of our dataset is related to the definition of a visual element. Can we consider a group of small elements, in close proximity to one another and representing a similar topic, as a single visual element, or should we label each of these small elements as individual visual elements? The importance of this question lies in the fact that our labelling technique naturally affects the desired model prediction output. In order to find a *historically* valid solution, we opted to divide such groups into smaller visual elements when it was possible to identify that they were printed using different woodblocks. These woodblocks were generally expensive and often re-used by different printers over a long period of time [52], which would mean not only that our labelling logic in this case is historically grounded, but that once correctly labelled, the same group of images are likely to repeat in other *Sphaera* editions in the same form. Despite this labelling methodology, errors persist where multiple small visual elements are grouped together by Cor*Deep* rather than classified as single visual elements as desired (see Figure 7 (right)).

The data itself, in this case S-VED, clearly dictates what the model detects. Since the Cor*Deep* model is trained on S-VED${}_{extended}$, the Cor*Deep* visual element detector usually results in effective detections in corpora of similar nature to the *Sphaera* Corpus. However, as shown in Section 5.2, the models possess relatively good generalization abilities, and can extract elements from out-of-domain corpora with high AP.

## 8. Conclusions

This paper presents two important contributions to traditional and computational historians alike. The first is the S-VED, which adds a relatively large, multi-class, historical visual-element-oriented dataset to the current repertoire of open access datasets and opens the door towards a fine-grained distant reading of large corpora through their visual elements. The second is Cor*Deep*, our open access web-service that allows any historian, without any prior machine learning knowledge, to seamlessly extract visual elements from entire corpora at the click of a button. The accessibility of this web-service, along with its historically oriented model, allow historians to investigate the content of large historical corpora by extracting their visual elements in distinct classes; a task that would otherwise require countless hours. The fact that the field of Digital Humanities is developing at lightning speed only emphasizes the importance of the above contributions and highlights the need to better disseminate historical datasets, as well as to provide easy-to-use computational models for general use [53]. These contributions are envisioned to be the first of many, while the next steps will focus on adding new classes to Cor*Deep*, allowing historians to detect not only visual elements, but also for instance numerical tables. In addition, larger versions of the S-VED are expected to be published once copyright conflicts are resolved, adding more visual elements to an already sizeable open access dataset.

## Figures and Tables

**Table 1:** The total number of pages, pages without any visual elements, and samples per class in S-VED and S-VED${}_{extended}$.

	S-VED	S-VED${}_{extended}$
**Total Pages**	4000	46380
**Pages w/o visual elements**	1960	23,190
**Content Illustrations**	2076	21,000
**Printer’s Marks**	19	235
**Decorations**	218	2041
**Initials**	614	6258

**Table 2:** Average precision (AP) results for both models for two different image input resolutions.

Model	S-VED (1280 × 1280)	S-VED(640 × 640)	S-VED${}_{extended}$ (1280 × 1280)	S-VED${}_{extended}$(640 × 640)
YOLOv5	0.962	0.974	0.983	**0.986**
Faster-RCNN	0.949	0.940	0.975	0.965
DocExtractor	0.890	0.735	0.925	0.809

**Table 3:** Average precision results for the four different types of visual elements as achieved by the YOLOv5 model with its best performing input image resolution 640 × 640 and the Faster-RCNN model with its best performing input image resolution 1280 × 1280. The mean average precisions are reported as the standard mean (mAP) over the APs of the classes and the weighted mean (wmAP), where APs are weighed according to the relative number of instances of the class.

	AP YOLOv5		AP Faster-RCNN	
**Class**	**S-VED**	**S-VED${}_{extended}$**	**S-VED**	**S-VED${}_{extended}$**
Content Illus.	0.976	0.988	0.955	0.978
Initials	0.987	0.988	0.981	0.987
Printer’s Marks	0.819	0.923	0.168	0.530
Decorations	0.834	0.92	0.592	0.899
	mAP = 0.904	mAP = 0.955	mAP = 0.674	mAP = 0.849
	wmAP = 0.969	wmAP = 0.984	wmAP = 0.934	wmAP = 0.971

**Table 4:** Performance of YOLOv5 trained on S-VED, S-VED${}_{extended}$, and SynDoc respectively with respect to the different historical document datasets (* marks results where recognized Initials are ignored).

Model Architecture	Trained on	Tested on	AP (1280 × 1280)	AP (640 × 640)
YOLOv5	S-VED	S-VED${}_{extended}$	0.962	0.974
		illuHisDoc	0.543	0.761
		RASM	0.474	0.762
		mandragore	0.228/0.617 *	0.312/0.509 *
		Pseudo Proclus	0.975	0.979
YOLOv5	S-VED${}_{extended}$	S-VED${}_{extended}$	0.983	**0.986**
		illuHisDoc	0.693	0.793
		RASM	0.725	**0.849**
		Mandragore	0.304/0.704 *	**0.416**/0.726 *
		Pseudo Proclus	0.985	**0.988**
YOLOv5	SynDoc	S-VED${}_{extended}$	0.787	0.807
		illuHisDoc	**0.826**	0.762
		RASM	0.714	0.714
		Mandragore	0.349	0.405
		Pseudo Proclus	0.905	0.788

**Table 5:** Cross validation results, where the YOLOv5 model was trained on all five data sets except the respective test set. A resolution of 640 × 640 was used for all data sets.

Trained on	Tested on	AP
S-VED, SynDoc, mandragore, RASM, Ps.Proclus	illuHisDoc	0.864
S-VED, SynDoc, illuHisDoc, mandragore, Ps.Proclus	RASM	0.961
S-VED, SynDoc, illuHisDoc, RASM, Ps.Proclus	mandragore	0.77
S-VED, SynDoc, illuHisDoc, RASM, mandragore	Pseudo Proclus	0.965
SynDoc, illuHisDoc, RASM, mandragore, Ps.Proclus	S-VED	0.948

## Data Availability

The data presented in this study are openly available in https://gitlab.gwdg.de/MPIWG/Department-I/sphaera/s-ved-object-detection, accessed on 2 October 2022.
